# Correction: MERS-CoV spillover at the camel-human interface

**DOI:** 10.7554/eLife.37324

**Published:** 2018-04-19

**Authors:** Gytis Dudas, Luiz Max Carvalho, Andrew Rambaut, Trevor Bedford

Dudas G, Carvalho LM, Rambaut A, Bedford T. 2018. MERS-CoV spillover at the camel-human interface. *eLife* **7**:e31257. doi: 10.7554/eLife.31257.

Published 16, January 2018

We noticed that a number of important citations were omitted in the original article. We have now corrected the article to add citations to give credit to the primary data producers without whose efforts our study would not have been possible, as well as giving credit to authors whose findings we unfortunately stated as self-evident.

Citations have been added to the following sentences in the “Sequence data” section of the Materials and methods as follows:

Original:

All MERS-CoV sequences were downloaded from GenBank and accession numbers are given in Supplementary File 1. Sequences without accessions were kindly shared by Ali M. Somily, Mazin Barry, Sarah S. Al Subaie, Abdulaziz A. BinSaeed, Fahad A. Alzamil, Waleed Zaher, Theeb Al Qahtani, Khaldoon Al Jerian, Scott J.N. McNabb, Imad A. Al-Jahdali, Ahmed M. Alotaibi, Nahid A. Batarfi, Matthew Cotten, Simon J. Watson, Spela Binter, and Paul Kellam prior to publication.

Corrected:

All MERS-CoV sequences were downloaded from GenBank and accession numbers are given in Supplementary File 1 (Assiri et al., 2016a,b; Azhar et al., 2014; van Boheemen et al., 2012; Briese et al., 2014; Chu et al., 2014; Cotten et al., 2013, 2014; Drosten et al., 2013, 2015; Fagbo et al., 2015; Haagmans et al., 2014; Hemida et al., 2014; Hunter et al., 2016; Kandeil et al., 2016; Kapoor et al., 2014; Kim et al., 2015, 2016; Lamers et al., 2016; Lau et al., 2016; Lu et al., 2017; Park et al., 2016a,b; Plipat et al., 2017; Raj et al., 2014; Sabir et al., 2016; Seong et al., 2016; Xie et al., 2015). Sequences without accessions were kindly shared by Ali M. Somily, Mazin Barry, Sarah S. Al Subaie, Abdulaziz A. BinSaeed, Fahad A. Alzamil, Waleed Zaher, Theeb Al Qahtani, Khaldoon Al Jerian, Scott J.N. McNabb, Imad A. Al-Jahdali, Ahmed M. Alotaibi, Nahid A. Batarfi, Matthew Cotten, Simon J. Watson, Spela Binter, and Paul Kellam prior to publication. Sequences available on GenBank but not associated with publications were shared by Meriadeg Ar Gouilh, Emad M. Elassal, Monica Galiano, Krista Queen and Ying Tao.

Citations have also been added to the following sentence in the “Recombination shapes MERS-CoV diversity” section of the Results as follows:

Original text:

... allowing for co-infection with distinct genotypes and thus recombination to occur."

Corrected text:

... allowing for co-infection with distinct genotypes and thus recombination to occur (Sabir et al, 2016)."

In connection with the changes above, the following citations have been added to the Reference list:

Assiri AM, Biggs HM, Abedi GR, Lu X, Bin Saeed A, Abdalla O, Mohammed M, Al-Abdely HM, Algarni HS, Alhakeem RF, Almasri MM, Alsharef AA, Nooh R, Erdman DD, Gerber SI, Watson JT. 2016. Increase in Middle East Respiratory Syndrome-Coronavirus Cases in Saudi Arabia Linked to Hospital Outbreak With Continued Circulation of Recombinant Virus, July 1–August 31, 2015. Open Forum Infectious Diseases 3(3): ofw165. doi: https://doi.org/10.1093/ofid/ofw165

Assiri AM, Midgley CM, Abedi GR, Bin Saeed A, Almasri MM, Lu X, Al-Abdely HM, Abdalla O, Mohammed M, Algarni HS, Alhakeem RF, Sakthivel SK, Nooh R, Alshayab Z, Alessa M, Srinivasamoorthy G, AlQahtani SY, Kheyami A, HajOmar WH, Banaser TM, et al. 2016. Epidemiology of a Novel Recombinant Middle East Respiratory Syndrome Coronavirus in Humans in Saudi Arabia. The Journal of Infectious Diseases 214(5):712-721. doi: https://doi.org/10.1093/infdis/jiw236

Azhar EI, El-Kafrawy SA, Farraj SA, Hassan AM, Al-Saeed MS, Hashem AM, Madani TA. 2014. Evidence for Camel-to-Human Transmission of MERS Coronavirus. New England Journal of Medicine 370(26):2499-2505. doi: 10.1056/NEJMoa1401505.

Briese T, Mishra N, Jain K, Zalmout IS, Jabado OJ, Karesh WB, Daszak P, Mohammed OB, Alagaili AN, Lipkin WI. 2014. Middle East Respiratory Syndrome Coronavirus Quasispecies That Include Homologues of Human Isolates Revealed through Whole-Genome Analysis and Virus Cultured from Dromedary Camels in Saudi Arabia. mBio 5(3):e01146-14. doi: https://doi.org/10.1128/mBio.01146-14

Cotten M, Watson SJ, Zumla AI, Makhdoom HQ, Palser AL, Ong SH, Rabeeah AAA, Alhakeem RF, Assiri A, Al-Tawfiq JA, Albarrak A, Barry M, Shibl A, Alrabiah FA, Hajjar S, Balkhy HH, Flemban H, Rambaut A, Kellam P, Memish ZA. 2014. Spread, Circulation, and Evolution of the Middle East Respiratory Syndrome Coronavirus. mBio 5(1):e01062–13. doi: https://doi.org/10.1128/mBio.01062-13

Drosten C, Seilmaier M, Corman VM, Hartmann W, Scheible G, Sack S, Guggemos W, Kallies R, Muth D, Junglen S, Müller MA, Haas W, Guberina H, Röhnisch T, Schmid-Wendtner M, Aldabbagh S, Dittmer U, Gold H, Graf P, Bonin F, et al. 2013. Clinical features and virological analysis of a case of Middle East respiratory syndrome coronavirus infection. The Lancet Infectious Diseases 13(9):745-751. doi: https://doi.org/10.1016/S1473-3099(13)70154-3

Drosten C, Muth D, Corman VM, Hussain R, Al Masri M, HajOmar W, Landt O, Assiri A, Eckerle I, Al Shangiti A, Al-Tawfiq JA, Albarrak A, Zumla A, Rambaut A, Memish ZA. 2015. An Observational, Laboratory-Based Study of Outbreaks of Middle East Respiratory Syndrome Coronavirus in Jeddah and Riyadh, Kingdom of Saudi Arabia, 2014. Clinical Infectious Diseases 60(3):369-377. doi: https://doi.org/10.1093/cid/ciu812

Haagmans BL, Al Dhahiry SHS, Reusken CBEM, Raj VS, Galiano M, Myers R, Godeke GJ, Jonges M, Farag E, Diab A, Ghobashy H, Alhajri F, Al-Thani M, Al-Marri SA, Al Romaihi HE, Al Khal A, Bermingham A, Osterhaus ADME, AlHajri MM, Koopmans MPG. 2014. Middle East respiratory syndrome coronavirus in dromedary camels: an outbreak investigation. The Lancet Infectious Diseases 14(2):140-145. doi: https://doi.org/10.1016/S1473-3099(13)70690-X

Hemida MG, Chu DKW, Poon LLM, Perera RAPM, Alhammadi MA, Ng Hy, Siu LY, Guan Y, Alnaeem A, Peiris M. 2014. MERS Coronavirus in Dromedary Camel Herd, Saudi Arabia. Emerging Infectious Diseases 20(7):1231-1234. doi: https://doi.org/10.3201/eid2007.140571

Hunter JC, Nguyen D, Aden B, Al Bandar Z, Al Dhaheri W, Abu Elkheir K, Khudair A, Al Mulla M, El Saleh F, Imam- baccus H, Al Kaabi N, Sheikh FA, Sasse J, Turner A, Abdel Wareth L, Weber S, Al Ameri A, Abu Amer W, Alami NN, Bunga S, et al. 2016. Transmission of Middle East Respiratory Syndrome Coronavirus Infections in Healthcare Settings, Abu Dhabi. Emerging Infectious Diseases 22(4):647-656. doi: https://doi.org/10.3201/eid2204.151615

Kandeil A, Shehata MM, El Shesheny R, Gomaa MR, Ali MA, Kayali G. 2016. Complete Genome Sequence of Middle East Respiratory Syndrome Coronavirus Isolated from a Dromedary Camel in Egypt. Genome Announcements 4(2). doi: https://dx.doi.org/10.1128/genomeA.00309-16

Kapoor M, Pringle K, Kumar A, Dearth S, Liu L, Lovchik J, Perez O, Pontones P, Richards S, Yeadon-Fagbohun J, Breakwell L, Chea N, Cohen NJ, Schneider E, Erdman D, Haynes L, Pallansch M, Tao Y, Tong S, Gerber S, et al. 2014. Clinical and Laboratory Findings of the First Imported Case of Middle East Respiratory Syndrome Coronavirus to the United States. Clinical Infectious Diseases 59(11):1511–1518. doi: https://doi.org/10.1093/cid/ciu635

Kim YJ, Cho YJ, Kim DW, Yang JS, Kim H, Park S, Han YW, Yun Mr, Lee HS, Kim AR, Heo DR, Kim JA, Kim SJ, Jung HD, Kim N, Yoon SH, Nam JG, Kang HJ, Cheong HM, Lee JS, et al. 2015. Complete Genome Sequence of Middle East Res- piratory Syndrome Coronavirus KOR/KNIH/002_05_2015, Isolated in South Korea. Genome Announcements 3(4). doi: https://doi.org/10.1128/genomeA.00787-15

Kim Y, Cheon S, Min CK, Sohn KM, Kang YJ, Cha YJ, Kang JI, Han SK, Ha NY, Kim G, Aigerim A, Shin HM, Choi MS, Kim S, Cho HS, Kim YS, Cho NH. 2016. Spread of Mutant Middle East Respiratory Syndrome Coronavirus with Reduced Affinity to Human CD26 during the South Korean Outbreak. mBio 7(2):e00019-16. doi: https://doi.org/10.1128/mBio.00019-16

Lamers MM, Raj VS, Shafei M, Ali SS, Abdallh SM, Gazo M, Nofal S, Lu X, Erdman DD, Koopmans MP, Abdallat M, Haddadin A, Haagmans BL. 2016. Deletion Variants of Middle East Respiratory Syndrome Coronavirus from Humans, Jordan, 2015. Emerging infectious diseases 22(4):716-719. doi: https://doi.org/10.3201/eid2204.152065

Lau SKP, Wernery R, Wong EYM, Joseph S, Tsang AKL, Patteril NAG, Elizabeth SK, Chan KH, Muhammed R, Kinne J, Yuen KY, Wernery U, Woo PCY. 2016. Polyphyletic origin of MERS coronaviruses and isolation of a novel clade A strain from dromedary camels in the United Arab Emirates. Emerging Microbes & Infections 5(12):e128. doi: https://doi.org/10.1038/emi.2016.129

Lu X, Rowe LA, Frace M, Stevens J, Abedi GR, Elnile O, Banassir T, Al-Masri M, Watson JT, Assiri A, Erdman DD. 2017. Spike gene deletion quasispecies in serum of patient with acute MERS-CoV infection. Journal of Medical Virology 89(3):542-545. doi: https://doi.org/10.1002/jmv.24652

Park D, Huh HJ, Kim YJ, Son DS, Jeon HJ, Im EH, Kim JW, Lee NY, Kang ES, Kang CI, Chung DR, Ahn JH, Peck KR, Choi SS, Kim YJ, Ki CS, Park WY. 2016. Analysis of intrapatient heterogeneity uncovers the microevolution of Middle East respiratory syndrome coronavirus. Cold Spring Harbor Molecular Case Studies 2(6). doi: https://doi.org/10.1101/mcs.a001214

Park WB, Kwon NJ, Choe PG, Choi SJ, Oh HS, Lee SM, Chong H, Kim JI, Song KH, Bang JH, Kim ES, Kim HB, Park SW, Kim NJ, Oh Md. 2016. Isolation of Middle East Respiratory Syndrome Coronavirus from a Patient of the 2015 Korean Outbreak. Journal of Korean Medical Science 31(2):315–320. doi: https://doi.org/10.3346/jkms.2016.31.2.315

Plipat T, Buathong R, Wacharapluesadee S, Siriarayapon P, Pittayawonganon C, Sangsajja C, Kaewpom T, Petcharat S, Ponpinit T, Jumpasri J, Joyjinda Y, Rodpan A, Ghai S, Jittmittraphap A, Khongwichit S, Smith DR, Corman VM, Drosten C, Hemachudha T. 2017. Imported case of Middle East respiratory syndrome coronavirus (MERS-CoV) infection from Oman to Thailand, June 2015. Eurosurveillance 22(33). doi: https://doi.org/10.2807/1560-7917.ES.2017.22.33.30598

Raj VS, Farag EABA, Reusken CBEM, Lamers MM, Pas SD, Voermans J, Smits SL, Osterhaus ADME, Al-Mawlawi N, Al-Romaihi HE, AlHajri MM, El-Sayed AM, Mohran KA, Ghobashy H, Alhajri F, Al-Thani M, Al-Marri SA, El-Maghraby MM, Koopmans MPG, Haagmans BL. 2014. Isolation of MERS coronavirus from a dromedary camel, Qatar, 2014. Emerging Infectious Diseases 20(8):1339-1342. doi: https://doi.org/10.3201/eid2008.140663

Sabir JSM, Lam TTY, Ahmed MMM, Li L, Shen Y, Abo-Aba SEM, Qureshi MI, Abu-Zeid M, Zhang Y, Khiyami MA, Alharbi NS, Hajrah NH, Sabir MJ, Mutwakil MHZ, Kabli SA, Alsulaimany FAS, Obaid AY, Zhou B, Smith DK, Holmes EC, et al. 2016. Co-circulation of three camel coronavirus species and recombination of MERS-CoVs in Saudi Arabia. Science 351(6268):81-84. doi: https://doi.org/10.1126/science.aac8608

Seong MW, Kim SY, Corman VM, Kim TS, Cho SI, Kim MJ, Lee SJ, Lee JS, Seo SH, Ahn JS, Yu BS, Park N, Oh Md, Park WB, Lee JY, Kim G, Joh JS, Jeong I, Kim EC, Drosten C, et al. 2016. Microevolution of Outbreak-Associated Middle East Respiratory Syndrome Coronavirus, South Korea, 2015. Emerging Infectious Diseases 22(2):327-330. doi: https://doi.org/10.3201/eid2202.151700

Xie Q, Cao Y, Su J, Wu X, Wan C, Ke C, Zhao W, Zhang B. 2015. Genomic sequencing and analysis of the first imported Middle East Respiratory Syndrome Coronavirus (MERS CoV) in China. Science China Life Sciences 58(8):818-820. doi: https://doi.org/10.1007/s11427-015-4903-7

We apologise for the omission of these citations.

The article has been corrected accordingly.

